# Does Neonatal Sepsis Independently Increase Neurodevelopmental Impairment?

**DOI:** 10.3390/children9040568

**Published:** 2022-04-16

**Authors:** Vishwanath Bhat, Vineet Bhandari

**Affiliations:** Division of Neonatology, The Children’s Regional Hospital at Cooper, Cooper Medical School of Rowan University, One Cooper Plaza, Camden, NJ 08103, USA; bhat-vishwanath@cooperhealth.edu

**Editorial Comment on**: Short- and long- term neurodevelopmental outcomes of very preterm infants with neonatal sepsis: a systematic review and meta-analysis. *Children***2019**, *6*, 131. http://doi.org/110.3390/children6120131

Globally, sepsis affects an estimated 3 million newborn infants annually, with mortality rates between 11% and 19% [1,2]. Despite this huge burden of disease, a consensus definition of neonatal sepsis is currently lacking [2,3], leading to considerable variability in the definitions used by both researchers and clinicians alike. The term neonatal sepsis refers to a systemic infection of bacterial, viral or fungal origin that is associated with hemodynamic changes and other clinical manifestations of organ dysfunction and is traditionally diagnosed (“gold standard”) by isolation of the pathogen from the blood or cerebrospinal fluid (CSF) [4]. The incidence of neonatal sepsis is inversely related to birth weight and gestational age [5,6,7]. Based on the age of onset of clinical manifestations, sepsis in very low birth weight (VLBW) infants has been classified as either early-onset sepsis (EOS), occurring within the first 72 h of life, or late-onset sepsis (LOS), with onset after 72 hours of life until hospital discharge [4,8]. EOS occurs due to vertical transmission of the organisms from the mother to the infant before or during delivery. Over the past 3 decades, there has been a shift from Group B *Streptococcus* (GBS) to *E. coli* as the most frequent pathogen causing EOS in VLBW infants [9,10]. Recent data from the National Institute of Child Health and Human Development (NICHD) Neonatal Research Network (NRN) and Vermont Oxford Network (VON) show that *E. coli* now accounts for nearly half (47–58%) of all cases of EOS among VLBW infants, followed by GBS (12–19%), *Hemophilus* species (8%) and *Staphylococcus aureus* (6%) [7,10]. LOS is caused by pathogens acquired via healthcare-associated transmission during the hospital course, or less frequently, by pathogens acquired at the time of birth with late manifestation. These pathogens include coagulase-negative *Staphylococcus* (CONS) (28%), Gram-negative organisms (26%) and *Staphylococcus aureus* (15%) [11].

Recent data from the NICHD NRN and VON show that the incidence of EOS among VLBW infants has changed little over the past two decades. Despite an increase in maternal antibiotic use during labor, the overall rate of EOS remains at approximately 1.4% of all VLBW infants [7,10]. However, the rates of LOS have seen a steady decline over the past two decades. Data from the 756 VON member neonatal intensive care units (NICUs) indicate a reduction in the mean risk-adjusted rates of LOS in VLBW infants from 20.6% in 2005 to 9.7% in 2014 [12].

The diagnosis of sepsis is especially challenging in VLBW/preterm neonates because of three primary issues: (i) hemodynamic changes and other clinical manifestations of organ dysfunction (e.g., respiratory distress, apnea, hypotension, feed intolerance, and temperature instability) are non-specific, and may be related to organ immaturity and/or pro-inflammatory cytokines); (ii) the concern for falsely negative blood cultures (type II error) due to inadequate blood sample volume or exposure of the infant to antibiotics prior to obtaining the blood sample or due to a viral infection; and (iii) the issue of false-positive blood cultures (type I error) due to contaminants, especially with CONS.

Short- and long-term neurodevelopmental outcomes are one of the most important benchmarks of neonatal care. Although the association between neonatal white matter injury on autopsy and bacteremia was first described almost five decades ago [13], the mechanisms underlying adverse neurodevelopmental outcomes following neonatal sepsis remain incompletely understood. In addition to the well-recognized cerebral white matter injury, recent advanced MRI techniques including volumetric data reveal pronounced deficits in cerebral cortical and deep nuclear gray matter volumes at term equivalent and during childhood among preterm infants [14]. The encephalopathy of prematurity includes these neuronal–axonal abnormalities in combination with white matter injury [14]. In infants with meningitis, brain injury may be caused by bacterial invasion of the brain, leading to direct cytotoxic injury and activation of local inflammatory response [15]. Preterm infants with a positive blood culture had a two-fold increase in the risk for white matter injury and those with positive CSF culture had a four-fold increase in the risk for white matter injury [16].

In this journal, Cai et al. [17] have presented a systematic review and meta-analysis to provide an updated summary of data from observational studies and address the important issue of short- and long- term neurodevelopmental outcomes of very preterm infants with neonatal sepsis. Of the 1166 articles identified through searching of database and reference lists, 24 articles were of sufficient quality to be included in the report, and only 14 observational studies, consisting of case–control and cohort studies, met the criteria for inclusion in the meta-analysis. Explicit selection criteria were used, including BW, gestational maturity, blood culture-proven sepsis and follow-up duration. The results of the meta-analysis demonstrated that very preterm infants with neonatal sepsis had significantly higher rates of neuro-developmental impairment (NDI) compared with these without sepsis (OR 3.18, 95% CI 2.29–4.41).

Unfortunately, only four studies reported a longer follow-up duration of ≥36 months. A subgroup analysis of these four studies showed a similar association (OR 3.07, 95% CI 1.79–5.28). As the authors acknowledge, there was substantial heterogeneity between studies in terms of study design (case–control vs. cohort studies), definitions of sepsis (positive blood culture with or without symptoms, meningitis, and/or inflammatory markers), causative pathogens (all pathogens vs. restricted to specific pathogens, e.g., *Candida*, CONS or Gram-negative pathogens), duration of follow-up, definition of NDI, and outcome measure (dichotomous vs. continuous). The five studies that reported outcomes as continuous variables showed no significant difference in the developmental scores between the sepsis and non-sepsis groups.

Since all included studies were non-randomized by design, the results of the individual studies would be significantly influenced by confounding factors, including gestational age, sex, BW, multiple births, mode of delivery, pre- and post- natal corticosteroid exposure, intrauterine growth restriction, chorioamnionitis, bronchopulmonary dysplasia, sonographic parenchymal brain injury, necrotizing enterocolitis and maternal education. The authors used unadjusted ORs to pool data for their meta-analysis since the individual studies adjusted differently to these confounders, which makes the interpretation of the pooled estimate of the effect size less reliable.

The risk of bias in individual studies was appropriately evaluated by the authors of this meta-analysis. The asymmetry of the contour-enhanced funnel plot indicates a high likelihood of publication or reporting bias, though the Egger’s meta-regression test suggests otherwise. The retrospective study design introduces the risk of selection bias. The results of follow-up studies are impacted by the proportion of study patients that are evaluated for the outcome measures. The wide variability in follow-up rates (55–97%) leads to a risk of attrition bias, since some studies have shown that patients who returned for follow-up had higher rates of infection. Detection bias and reporting bias are also likely since blinding of the personnel performing the developmental evaluation was not reported in a majority of the studies.

Given that the included studies were heterogeneous, non-randomized and influenced by several confounding factors, and that unadjusted ORs were used, sensitivity analysis would have been helpful to evaluate the role of selection bias (prospective cohort studies only), attrition bias (studies with high follow-up rates), or relative contribution of specific pathogens (studies involving *Candida* sepsis or CONS sepsis). The differences in neurodevelopmental outcomes following EOS versus LOS is also unclear. Infants with late bacteremia have been shown to have a much stronger and more sustained inflammatory response compared to infants with early bacteremia [18].

These results are consistent with the results of a previous meta-analysis by Alshaikh et al. published in 2013 [19]. In this meta-analysis of 17 studies, with age at follow-up ranging from 6 months to 60 months, sepsis in VLBW infants was associated with a two-fold increase in NDI.

Since the publication of the meta-analysis by Cai et al., several studies have confirmed the association between neonatal infection and NDI, but with much smaller effect sizes [20,21,22,23]. In a recent large retrospective cohort study of 6565 infants with birth weights of 401–1000 grams born at NICHD NRN centers, Mukhopadhyay et al. showed that infants with culture-positive EOS were significantly more likely to have death/NDI (adjusted relative risk (aRR) 1.23, 95% CI 1.10–1.37) and NDI (aRR 1.34, 95% CI 1.05–1.71), compared to unaffected infants [20]. In another study by the same group of investigators, involving 3940 infants with birth weights of 401–1000 g, infants with culture-positive LOS were significantly more likely to have death/NDI (aRR 1.29, 95% CI 1.17–1.42) but not NDI alone (aRR 1.15, 95% CI 0.99–1.34), compared to unaffected infants [22]. A prospective cohort study involving over 2000 infants enrolled in the Korean Neonatal Network born at 23–32 weeks of gestation, demonstrated a significant association of LOS with cognitive delay (OR 1.48, 95% CI 1.02–2.16) but no association with motor delay at 18–24 months of corrected age [23].

The strength of any meta-analysis is largely dependent upon the quality of the included studies. This study uncovers the challenges of summarizing data that are heterogeneous secondary to multiple largely unavoidable factors. Additionally, it underscores the need to establish a consensus definition for neonatal sepsis, which would not only facilitate the comparison of outcomes across centers, but also lead to improved quality of clinical care. The authors ought to be commended for highlighting an extremely relevant and important subject. Due to the methodologic limitations, including the paucity of high-quality research and heterogeneity across studies as reported by the authors, one needs to interpret the results of this meta-analysis with caution.

Given that all these studies are observational in nature, a causal inference between infection and neurodevelopmental outcomes cannot be made. It is possible that an altered gut microbiome may act as a common predisposing factor for both infection and neurodevelopmental impairment [24,25], although definitive studies are lacking. The potential effects of the choice and duration of antibiotics on the gut microbiome and developmental outcomes also remain unknown.
